# Monoclonal antibodies binding to different epitopes of CD20 differentially sensitize DLBCL to different classes of chemotherapy

**DOI:** 10.3389/fonc.2023.1159484

**Published:** 2023-08-03

**Authors:** Brian Lee, Tim Pierpont, Avery August, Kristy Richards

**Affiliations:** ^1^ Department of Biochemistry and Molecular Genetics, Northwestern University Feinberg School of Medicine, Chicago, IL, United States; ^2^ Department of Microbiology and Immunology, College of Veterinary Medicine, Cornell University, Ithaca, NY, United States; ^3^ Department of Biomedical Sciences, College of Veterinary Medicine, Cornell University, Ithaca, NY, United States

**Keywords:** rituximab, ofatumumab, chemotherapy, R-CHOP, B-cell lymphoma, DLBCL

## Abstract

**Introduction:**

Rituximab (R), an anti-CD20 monoclonal antibody (mAb) and the world’s first approved antibody for oncology patients, was combined with the CHOP chemotherapy regimen and markedly improved the prognosis of all B- cell–derived lymphomas, the most common hematological malignancy worldwide. However, there is a 35% disease recurrence with no advancement in the first-line treatment since R was combined with the archetypal CHOP chemotherapy regimen nearly 30 years ago. There is evidence that R synergizes with chemotherapy, but the pharmacological interactions between R and CHOP or between newer anti-CD20 mAbs and CHOP remain largely unexplored.

**Methods:**

We used *in vitro* models to score pharmacological interactions between R and CHOP across various lymphoma cell lines. We compared these pharmacological interactions to ofatumumab, a second-generation anti-CD20 mAb, and CHOP. Lastly, we used RNA-sequencing to characterize the transcriptional profiles induced by these two antibodies and potential molecular pathways that mediate their different effects.

**Results:**

We discovered vast heterogeneity in the pharmacological interactions between R and CHOP in a way not predicted by the current clinical classification. We then discovered that R and ofatumumab differentially synergize with the cytotoxic and cytostatic capabilities of CHOP in separate distinct subsets of B-cell lymphoma cell lines, thereby expanding favorable immunochemotherapy interactions across a greater range of cell lines beyond those induced by R-CHOP. Lastly, we discovered these two mAbs differentially modulate genes enriched in the JNK and p38 MAPK family, which regulates apoptosis and proliferation.

**Discussion:**

Our findings were completely unexpected because these mAbs were long considered to be biological and clinical equivalents but, in practice, may perform better than the other in a patient-specific manner. This finding may have immediate clinical significance because both immunochemotherapy combinations are already FDA-approved with no difference in toxicity across phase I, II, and III clinical trials. Therefore, this finding could inform a new precision medicine strategy to provide additional therapeutic benefit to patients with B-cell lymphoma using immunochemotherapy combinations that already meet the clinical standard of care.

## Introduction

Non-Hodgkin’s lymphoma is the most common hematological malignancy worldwide, encompassing a heterogenous group of disorders that rank fifth highest in cancer mortality and seventh in cancer incidence (1). Rituximab (R) is the first monoclonal antibody (mAb) used in oncology treatment, and its addition to the CHOP [cyclophosphamide (C); doxorubicin (H), a topoisomerase II inhibitor; vincristine (O), an anti-microtubule drug; and prednisone (P), a glucocorticoid steroid] combination chemotherapy regimen doubled the average cure rates from approximately 30% to 60% of patients with non-Hodgkin’s B-cell lymphoma without increasing toxicity (2–6). R was subsequently combined with other chemotherapy regimens across all lymphoma subtypes, and these new immunochemotherapy combinations improved the prognosis of all B-cell–derived lymphoproliferative diseases (7–12). However, at least eight clinical trials utilizing either more intensive chemotherapy, small-molecule inhibitors, or newer mAbs have all generally failed to improve upon the R-CHOP regimen success (13–21). These combinations had either worse or similar clinical outcomes or higher toxic fatality rates. Therefore, despite over 20 years since R-CHOP was introduced, clinical protocol for the first-line treatment of diffuse large B-cell lymphoma (DLBCL) has remained virtually unchanged. R is an anti-CD20 antibody whose binding induces cell death through four different mechanisms: 1) direct signaling induced cell death, 2) complement-dependent cytotoxicity (CDC), 3) antibody-dependent cell-mediated cytotoxicity (ADCC), and 4) antibody-dependent phagocytosis (ADP) (22). Newer anti-CD20s, ofatumumab (OF) and obinutuzumab, which preferentially activate CDC and ADCC, respectively, were combined with conventional chemotherapy but did not improve prognosis in patients with DLBCL in their respective 2017 and 2019 phase III clinical trials compared with R-CHOP (18–21).

There is evidence that mAbs such as R sensitizes or confers resistance in B-cell cancers to chemotherapeutics through modulation of anti-apoptotic factors (23–25). Strategies that downregulate these anti-apoptotic factors may have a high therapeutic potential because this approach has already been applied to other cancers, leading to FDA-approved drugs such as venetoclax to treat leukemia (26). However, interactions between R and CHOP or between newer anti-CD20s and CHOP have not been studied, despite the potential that differential interactions could lead to better or worse outcomes depending on the mAb choice for specific patients. Furthermore, the direct mechanisms of cell death induced by anti-CD20s are poorly understood (22). Therefore, understanding how R and other anti-CD20 mAbs interact with CHOP can advise new therapeutic strategies and even help elucidate anti-CD20’s direct biological effects.

Here, we scored and characterized the pharmacological interactions between R and the individual components of CHOP. We then compared these interactions to those between OF and CHOP to identify any potential differential immunochemotherapy interactions. Last, we compared the transcriptional profiles of R and OF binding to mechanistically understand their differences.

## Materials and methods

### Cell lines and cell culture

Activated B-cell (ABC) subtypes (HBL1, U2932, SUDHL2, and TMD8) and germinal center B-cell (GCB) subtypes (SUDHL4, HT, LY18, and WSL-DLCL2) were maintained in Roswell Park Memorial Institute (RPMI) 1640 with 2 mM L-glutamine (Gibco), supplemented with 10% Fetal bovine serum (FBS) heat-inactivated (HI) at 56°C for 30 min (Gibco), penicillin (100 µg/mL) and streptomycin (100 µg/mL) (Gibco), and 1 mM N-2-hydroxyethylpiperazine-N-2-ethane sulfonic acid (HEPES) (Gibco). Cells were incubated at 37°C with 5% CO_2_ and maintained in a log growth phase. Cell viability and growth phase were measured using trypan blue exclusion assay, and cells were only used in a log growth phase with viability greater than 90% for all cell lines.

### Reagents

R (Rituxan™, Genentech, Inc.) and OF (Arzerra™, GlaxoSmithKline, Inc.) were obtained through the North Carolina Cancer Hospital pharmacy. Cyclophosphamide was replaced with its active metabolite 4-hydroperoxycyclophosphamide (Toronto Research Chemicals), which was dissolved in Dimethyl sulfoxide (DMSO) purged with nitrogen gas to make a stock concentration of 20 mM and sealed in vials with nitrogen gas at −80°C. Doxorubicin and vincristine (Selleckchem) were dissolved in Phosphate buffered saline (PBS) to make a stock concentration of 20 mM and were stored at −80°C. Prednisone was replaced with a more soluble biological and clinical analog, dexamethasone (Selleckchem), which was dissolved in PBS to make a stock concentration of 20 mM and stored at −80°C. We made this substitution because prednisone is metabolically converted to the more biologically active form prednisolone, in which the 11-C ketone is reduced to a more soluble 11-C hydroxyl, and dexamethasone has this more soluble substituent conferring its similar properties to prednisone in treating hematological malignancies (27). Pooled AB human serum (HS) was obtained from Sigma-Aldrich.

### Dose–response curves and analysis of pharmacological interactions

Survival dose–response curves for each of the CHOP drugs, with or without R, were generated to characterize the pharmacological interactions between R and CHOP. Cells were resuspended in fresh media the day before and seeded at 1.0 × 10^5^ cells/mL in 96-well flat bottom plates and treated for 48 h with varying concentrations of chemotherapeutic, with or without R (at 20 µg/mL) in triplicate. This concentration of R was used because pharmacokinetic studies have shown that R serum levels were, on average, 20.3 µg/mL 3 months after their infusion (28). Viability was measured using a CellTiter-Blue reduction assay by adding 10% CellTiter-Blue at 45.5 h after treatment and taking fluorescence readings at 540 nm/620 nm on a microplate reader (BioTek Synergy 2) at 46 and 48 h after treatment. Baseline fluorescent values at 46 h were subtracted from 48-h values to measure change in reduction potentials. By means of reduction potential, dose–response curves were made for each of the CHOP drugs with and without R (20 µg/mL) for two ABC subtypes (U2932 and HT) and two GCB subtypes (HBL1 and SUDHL4).

The chemotherapeutic-only dose–response curve was standardized to a medium-only control, and the R + chemo dose–response curve was standardized to an R-treated control by dividing fluorescent output of these two treatment groups by their respective controls. A 15% DMSO all-kill control was subtracted from both the numerator and denominator. Therefore, the viability was calculated by the following equations:


$$Viability\;\left(/control\right)=\frac{\Delta Fluorescence_{chemo}-\Delta Fluorescence_{DMSO}}{\Delta Fluorescence_{media}-\Delta Fluorescence_{DMSO}}$$

$$Viability\;\left(/control\right)=\frac{\Delta Fluorescence_{R+chemo}-\Delta Fluorescence_{DMSO}}{\Delta Fluorescence_{R}-\Delta Fluorescence_{DMSO}}$$

To design a dose–response curve with least squares fit, we transformed the drug concentrations to logarithmic scale and standardize viability to run between 0% and 100%. We then used the following equation to generate a sigmoidal regression fit into our dose–response data, where Hill slope coefficient is determined on the basis of the data


$$Viability=\frac{100}{1+10^{\text{log}\left(IC50-\left[drug\right]\times Hill\;Slope\right)}}$$

To assess the magnitude and statistical significance of these pharmacological interactions, we interpolated Half-maximal inhibitory concentration (IC_50_) values based on our nonlinear regression equation and their associated p-value using extra-sum-of-squares F-test. Last, we use the combination index analysis based on the Loewe additivity criteria and the response additivity analysis to score the pharmacological interactions as synergistic or antagonistic.

### Growth inhibition and cell death analysis

Trypan blue exclusion assay was used to characterize the pharmacological interactions observed in the experiments above. Cells were treated as described above and mixed with an equal volume of 0.4% trypan blue dye solution, and viability was determined by light microscopy. Percent dead was calculated as proportion of dead cells over total cells, whereas growth inhibition was calculated as total cells, both dead and alive, over total cells in medium-only conditions. The results were representative of three independent experiments.

### Complement-dependent cytotoxicity

**Figure 1:**
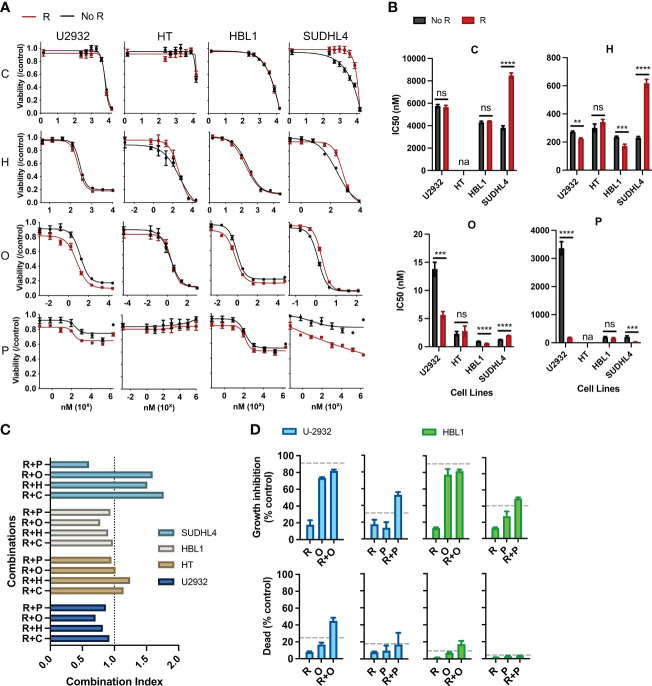
Rituximab directly synergizes and antagonizes the cytotoxic and cytostatic potential of chemotherapy. **(A)** Two ABC subtypes (HBL1 and SUDHL4) and two GCB subtypes (U2932 and HT) were treated with one of the CHOP drugs (0–15 µM C, 0–50 µM H, 0–0.125 µM O, and 0–1.8 × 10^3^ µM P) with or without R (20 µg/mL) for 48 h, and viability was measured using cell titer reduction assay. C_max_ for each drug are C (<27 µM), H (<1.1 µM), O (9.3 × 10^2^ µM), and P (3.1 × 10^3^ µM). No R (black curve) was standardized to a media control, whereas R (red curve) was standardized to R-treated group. This figure is representative of at least five experiments performed in triplicates. **(B)** IC_50_ of chemo and R + chemo combinations were also compared. *p < 0.05; **p < 0.01; ***p < 0.001; ****p < 0.0001. ns, not significant; na indicates that IC_50_ was unavailable due to dose–response curves not following sigmoidal relationship necessary for its calculation. **(C)** Combination index of each R + chemo combinations was scored. **(D)** HBL1 and U2932 cells were treated as indicated and trypan blue exclusion assay used to determine the percent of dead cells and percentage growth inhibition relative to a medium-only control. The dashed lines represent the expected additive effect of the sum of the individual drugs. This figure is representative of at least three experiments performed in triplicates.

CDC assays were performed using pooled human AB serum (Krackler Scientific) and R (20 µg/mL). Cell lines were incubated with varying proportions of HI FBS and HS totaling 10% net serum that induced approximately 50% cell death (10% HS and no HI FBS for HBL1 and HT, 5% HS and 5% HI FBS for U2932, and 2% HS and 8% HI FBS for SUDHL4). As a control, cells were incubated in HI HS (HI; 56°C for 30 min) and HI FBS in analogous concentrations. HI FBS was used to recapitulate growth conditions as similarly as possible to those used to generate dose–response curves and pharmacological analysis in **Figure 1**. To investigate the pharmacological interactions between CDC and chemotherapeutic, dose–response curves were created by treating cells with chemo as described above with or without CDC (HS + R + chemo and HS + chemo). As a control comparison, cells were also treated with HI + R + chemo and HI + chemo. Viability was measured using CellTiter-Blue as described above, and the pharmacological interactions were scored as described above on the basis of the relative curve shifts.

### Focused comparison between R + chemo and OF + chemo

Abbreviated experiments comparing differential interactions between R + chemo and OF + chemo were performed by treating all eight cell lines with physiologically relevant concentrations of C (<27 µM), H (<1.1 µM), O (9.3 × 10^−2^ µM), and P (3.1 × 10^3^ µM) based on the C_max_ of pharmacokinetic studies for each drug (29–32), with or without R or OF (10 µg/mL). Cell lines were treated for 48 h, and viability was measured using CellTiter-Blue as described earlier.

### RNA extraction and gene expression analysis

TMD8 and U2932 cells were split and cultured separately for a week to create two biological replicates per cell line. These replicates were then treated with R, OF, or human Immunoglobulin G1 (IgG1) isotype (SouthernBiotech) control for 4 h, followed by collection of RNA for sequencing. RNA from 8.0 × 10^7^ TMD8 and U2932 cells was extracted using the standard TRIzol protocol. Agarose gel (1%) was used to validate intact RNA by identifying the 28S and 18S ribosomal RNA bands. The library preparation, sequencing, and initial quality check were performed by the Cornell TREx sequencing facility (https://rnaseqcore.vet.cornell.edu/index.html). Specifically, fastq files are first processed with trim-galore to remove low quality bases and adapter sequences. Trimmed reads are then aligned to a human reference genome from Ensembl using STAR. Last, raw counts generated by STAR for annotated genes were analyzed with DESeq2.

### Reverse transcription/quantitative PCR

RNA was extracted using TRIzol reagent (Invitrogen) or an RNAeasy kit (Invitrogen), and Complementary DNA (cDNA) was generated using a You Prime First-beads kit (GE Healthcare). Quantitative PCR was then performed using a 7500 Fast Real-Time PCR instrument (Applied Biosystems). Data were analyzed using the comparative threshold cycle 2^−ΔΔCT^ method, and the values were expressed as fold change compared to respective cell lines treated with IgG-isotype antibody. Primer sequences for eight randomly selected genes are provided in **Table 1**.

**Table 1:** Validation of RNA sequencing (RNA-seq) using quantitative reverse transcription (qRT)-PCR.

Gene	Name	Primers	
ATP2A3	ATPase sarcoplasmic/endoplasmic reticulum Ca^2+^ transporting 3	Forward	5′-GCTCCAGATATCTCTGCCTGTC-3′
BCL2A1	BCL2-related protein A1	Forward	5′-TATGCTGGTAGAGTCAGTGGC-3′
		Reverse	5′-TATGCTGGTAGAGTCAGTGGC-3′
CDKN2C	Cyclin-dependent kinase inhibitor 2C	Forward	5′ATTTGGAAGGACTGCGCTGC-3′
		Reverse	5′-GCAGTCTCCTGGCAATCTCG-3′
DUSP5	Dual-specificity phosphatase A5	Forward	5′-CTGCAGCTCCTGTGGGAC-3′
		Reverse	5′-CACTGCCGAGGTAGAGGAAG-3′
RPSKA5	Ribosomal protein S6 kinase A5	Forward	5′-GGAGAGATTGTGCTTGCCCT-3′
		Reverse	5′-TCTGTCAGCACCACATGGC-3′
KCNMB4	Potassium calcium-activated channel subfamily M regulatory beta subunit 4	Forward	5′-GGTTCCCAGCCATTTACTTGC-3′
		Reverse	5′-CATGAGTGCGATGCAGAAGC-3′
NDFIP	Thioredoxin-interacting protein	Forward	5′-GGCAGCTGCTCATAGAACAAG-3′
		Reverse	5′-AAGGAATGTCGGGTTGATGC-3′
NLRC3	NLR family card protein containing 3	Forward	5′-TCGAGGCCCGGGAGAAC-3′
		Reverse	5′-GCGCCTTGGTGTCTTCATTTG-3′

### Pathway enrichment and visual analysis of differentially expressed genes

Kyoto Encyclopedia of Genes and Genomes (KEGG; www.genome.jp/kegg/pathway.html) pathway analysis was conducted to identify the most significant enriched pathways. Differentially expressed genes were mapped onto biologically relevant pathways using their manually annotated pathway database.

### Statistical analysis

All values are presented as mean ± standard deviation of the mean (SD) of data from experiments performed at least in triplicate. For comparisons between two groups, unpaired Student’s t-test was used. P-values for IC_50_’s were calculated using extra-sum-of-squares F-test. For multiple comparisons, one-way ANOVA was used with Bonferroni correction to adjust p-values based on the number of comparisons performed.

## Results

### Rituximab directly potentiates or antagonizes the cytotoxic and cytostatic potential of chemotherapy

R has been shown to affect chemotherapeutic responses separately from its immune effector functions (CDC, ADCC, and ADP), but such interactions within the R-CHOP regimen remain largely unexplored. Therefore, we scored the pharmacological interactions between R and the individual constituents of CHOP using CellTiter-Blue reduction assays, which measure the cumulative metabolic potential of living cells after treatment. We considered drug interactions in terms of potentiation and antagonism based on the criteria set by Gessner (33). Namely, we defined potentiation as a non-killing response that enhances killing by another drug and antagonism as a non-active response that reduces killing by another drug. We assigned R as the non-killing drug because previous studies, and our findings, show that R alone induces limited direct killing in most DLBCL cell lines, and this criterion was used to study the effects of R with other chemotherapy drugs (22, 34, 35).

Pharmacodynamic interactions between R and C, H, O, or P were first assessed in two ABC-subtype (U2932 and HT) and two GCB-subtype (HBL1 and SUDHL4) DLBCL. A viability dose–response curve was created with chemotherapy treatment along a concentration range spanning the entire drug effect. Another viability dose–response curve was created across the same concentration range with the addition of R (20 µg/mL). Cells were incubated for 48 h, and cell viability was measured using luminescent reduction assay (CellTiter-Blue). Chemo viability was normalized to untreated viability, whereas R + chemo viability was normalized to R-treated. This strategy removed any killing directly induced by R, thereby allowing for a direct comparison of the chemo-only and R + chemo dose–response curves for pharmacological interactions. Therefore, if the viability curve of R + chemo shifted to the right relative to chemo-only, then viability was higher with the addition of R to chemotherapy, indicating antagonism. If the two curves overlap, then there is no indication of interaction. Last, if the R + chemo viability curve shifted to the left relative to chemo-only, then viability is reduced with the addition of R, indicating a potentiation.

All chemotherapy drugs, with the exception of P, induced a significant loss of viability, with P having more modest effects (**Figure 1A**). Although P is cytotoxic to DLBCL in the first-line clinical care, the observed limited cytotoxicity in cell culture is consistent with other reported *in vitro* studies, likely reflecting selection for prednisone resistance through the establishment of cell lines from post-treatment patients (36). R potentiated O and P in HBL1 and U2932 but did not interact with chemotherapy in HT. R antagonized C, H, and O in SUDHL4 and potentiated P in SUDHL4 (**Figure 1A**). Therefore, R + chemo interactions were highly heterogenous, with different cell lines having differential responses to the same drugs, but not in a way predicted by the classical clinical classifications, namely, ABC and GCB subtypes. This heterogeneity in pharmacological interactions, ranging from antagonism to potentiation, was unexpected because R has been characterized as a general potentiator of chemotherapy in the field (22, 34, 35, 37). This heterogeneity in drug response illustrates the wide biological heterogeneity of DLBCL and possibly contributes to the variability in patient response to R-CHOP (38). To further quantify the magnitude and statistical significance of these pharmacological interactions, we compared the IC_50_’s of C, H, or O, with or without R (**Figure 1B**). In addition, to assess R + chemo interactions as synergistic or antagonistic, we use combination index analysis based on the Loewe additivity criteria (**Figure 1C**). Similar to our previous finding, R synergizes and antagonizes with chemotherapy in an ABC- and GCB-independent manner.

Our initial approach so far measured cell viability using CellTiter-Blue assay, which measures the cumulative metabolic reduction of living cells after treatment. Although this assay measures the total metabolism, and therefore viability, of the remaining cell population, it cannot differentiate potentiation and antagonism in terms of the cytotoxic or cytostatic effects. Therefore, we used a trypan blue exclusion assay, able to distinguish cell death from cell growth inhibition, to further characterize observed the pharmacological interactions in terms of the cytotoxic and cytostatic effects (34). We specifically examined interactions between R + O and R + P in HBL1 and U2932 cell lines because these combinations and their respective cell lines showed potentiation. R + O led to additive and greater than additive killing in HBL1 and U2932, respectively (**Figure 1D**). R + P induced greater than additive growth inhibition in both cell lines. Therefore, R can synergistically augment the cytotoxic and cytostatic potential of chemotherapy.

R also induces cell killing through other immune effector functions, and interactions between these immune mechanisms and chemotherapy remain unexplored in the R-CHOP regimen. Therefore, we investigated the interactions between R induced CDC and chemotherapy. Although we observed that R can kill DLBCL via CDC when HS was present versus when HI HS was present, we observed that CDC does not synergize with nor antagonize chemotherapy (**Supplementary Figure 1**).

### Rituximab and ofatumumab differentially potentiate chemotherapy in DLBCL

**Figure 2:**
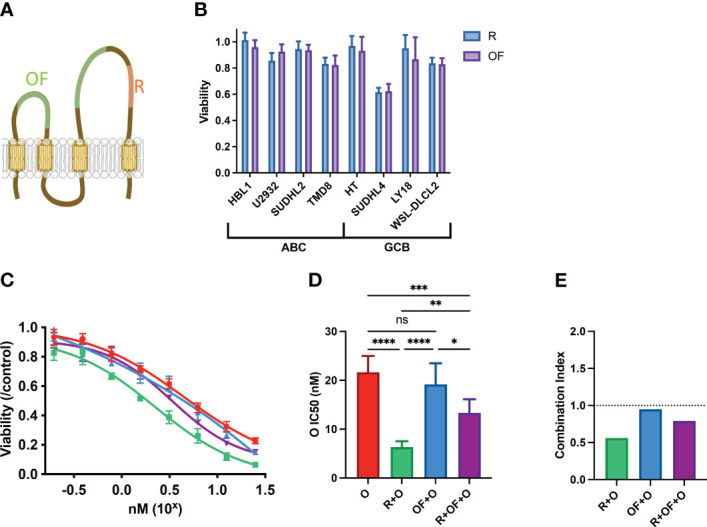
Rituximab and ofatumumab induce similar levels of direct killing but differentially synergize with chemotherapy. **(A)** Depiction of R and OF epitopes on CD20. **(B)** Direct killing induced by R and OF across eight cell lines. **(C)** Dose–response curve of O, R + O, OF + O, and R + OF + O. **(D)** IC_50_ of the effect of O, R + O, OF + O, and R + OF + O on U2932 cells. *p < 0.05; **p < 0.01; ***p < 0.001; ****p < 0.0001. ns, not significant. **(E)** Combination index of respective anti-CD20 + chemo combinations. This figure is representative of at least three experiments performed in triplicates.

There are no studies on the pharmacological interactions between newer anti-CD20 antibodies and CHOP. We therefore compared the pharmacological interactions between R + chemo and OF+ chemo to potentially identify any differences in immunochemotherapy interactions, which may elucidate molecular pathways that drive these immunochemotherapy interactions. R binds to the large loop on the extracellular domain of CD20, whereas OF binds an area distinct from the area bound by R, a region encompassing the small and large loop (**Figure 2A**). However, they both exhibit similar levels of direct killing and performed similarly in clinical trials (18–21). We determined their similarities *in vitro* via measurement of their direct killing on four ABC-subtype DLBCLs (HBL1, U2932, SUDHL2, andTMD8) and four GCB-subtype DLBCLs (HT, SUDHL4, LY18, and WSL-DLBCL2) and found that that they were comparable in their ability to reduce viability (**Figure 2B**). Although anti-CD20s usually induce limited direct killing, we note that R induces a significant viability reduction in SUDHL4.

Although R and OF seemed to induce similar direct effects, we compared their pharmacological interactions with chemotherapy. Because we previously observed that R potentiated chemotherapy in U2932, we treated U2932 with different doses of O alone, in combination with R and in combination with OF (**Figure 2C**). Unexpectedly, despite R and OF inducing similar levels of direct killing, they differentially potentiated killing by O. R potentiated O to a greater extent than OF. We also combined R + OF + O to determine whether R and OF together potentiate chemotherapy in an additive or synergistic manner (**Figure 2C**). We find that these two antibodies sensitized U2932 to chemotherapy intermediate between R + O and R + OF. These findings that R potentiated O in U2932, OF potentiated O to a more limited extent, and combining the two anti-CD20s with O led to an intermediate effect may suggest that R and OF interfere with one another for their respective CD20 epitope and that simply giving them both together would not compensate for any differential interactions with CHOP. This interference could possibly be due to the relatively small size of CD20, causing R and OF to sterically interfere with one another for their respective epitopes (39). To quantify the magnitude and the statistical significance of these differences, we compared the IC_50_’s of these of these immunochemotherapy interactions (**Figure 2D**). In addition, we use combination index analysis based on the Loewe additivity criteria and observe that R synergizes with chemotherapy, whereas OF does not (**Figure 2E**).

**Figure 3:**
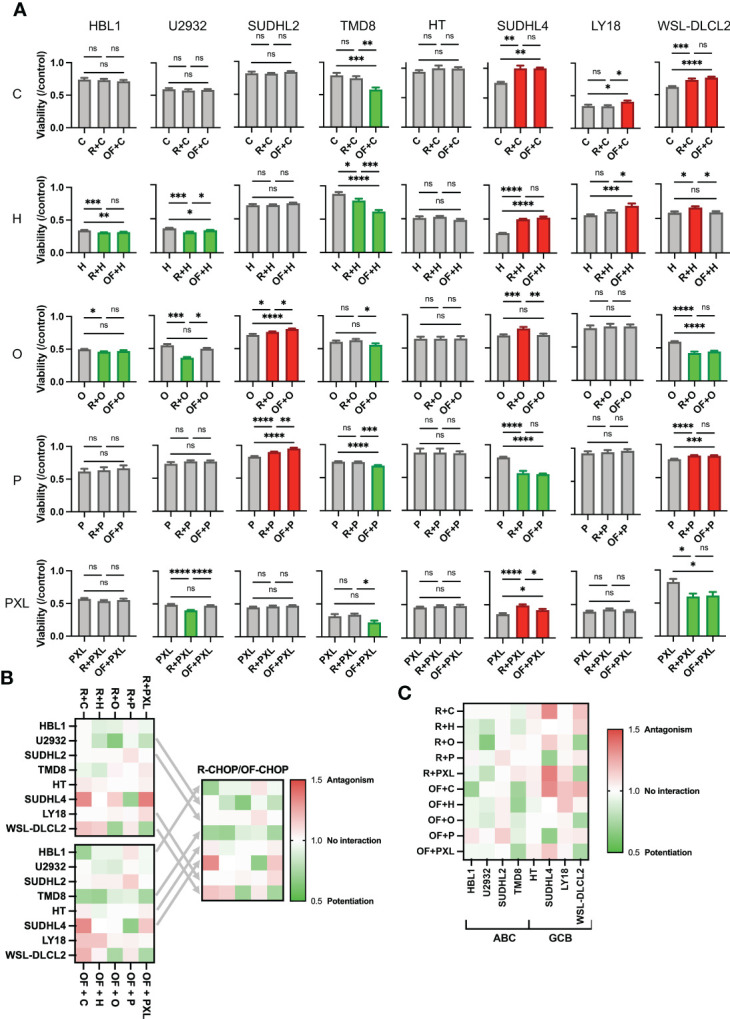
Rituximab and ofatumumab differentially synergize with chemotherapy, thereby expanding favorable immunochemotherapy interactions across a greater range of cell lines versus R-CHOP alone. **(A)** Comparison of the effect of R + CHOP versus OF + CHOP interactions across eight cell lines. For a given graph, the left bar represents viability of chemo-only, the middle bar represents R + chemo, and the right bar represents OF + chemo. Paclitaxel (PXL) was also tested. Green indicates potentiation, red indicates antagonism, and gray indicates no interaction. One-way ANOVA was used with p-value adjusted using Bonferroni corrections for multiple comparisons. *p < 0.05; **p < 0.01; ***p < 0.001; ****p < 0.0001. ns, not significant. This figure is representative of at least three experiments. **(B)** Heat map displaying combination indexes using R-CHOP only, OF-CHOP only, and cell line-tailored use of anti-CD20s with CHOP. **(C)** Heat map globally displaying combination indexes using either of the two mAbs with CHOP.

To determine whether this difference in pharmacological interaction was occurring in a U2932 cell–specific manner, all other seven cell lines mentioned previously were treated using a single physiologically relevant dose of one of the CHOP drugs independently or in combination with R or OF. Physiological concentrations were based on the C_max_ of each drug based on pharmacokinetic studies C (<27 µM), H (<1.1 µM), O (9.3 × 10^−2^ µM), and P (3.1 × 10^3^ µM) (29–32). R + Paclitaxel (PXL) was also examined as previous published studies demonstrated that R + PXL synergistically induces apoptosis (40). R + chemo and OF + chemo were compared to a chemo-only treatment group to score pharmacological interactions. R + chemo and OF + chemo were then compared to each other to score differential interactions between the two anti-CD20 mAbs. Although we found that R and OF induced comparable levels of direct killing, they unexpectedly potentiated chemotherapy differentially in six of eight cell lines (**Figure 3A**). R exhibited more favorable interactions with chemotherapy in U2932 (ABC), SUDHL2 (ABC), and LY18 (GCB), whereas OF exhibited more favorable chemotherapy interactions in TMD8 (ABC), SUDHL4 (GCB), and WSL-DLCL2 (GCB). Using the preferred anti-CD20 antibody along with chemotherapy reduced or eliminated antagonism and enhanced or induced potentiation with at least one of the CHOP drugs and all four in some cases. Therefore, tailored use of these anti-CD20 mAbs to each cell line expanded therapeutically favorable immunochemotherapy interactions across a greater range of cell lines than using R-CHOP or OF-CHOP alone. The expansion of synergy through cell line–specific use of these anti-CD20s with chemotherapy is illustrated through a heat map of the combination index of all pharmacological interactions (**Figure 3B**). Tailored use of these antibodies leads to expansion of synergy (green) and reduction in antagonism (red).

Last, a heat map of all pharmacological interactions using combination index analysis displays these interactions globally (**Figure 3C**). From this perspective, we observe that anti-CD20s preferentially synergize chemotherapy in the ABC subtype and antagonize chemotherapy in the GCB subtype.

### Comparison of gene expression profiles induced by rituximab and ofatumumab

Our finding that R and OF differentially synergize with CHOP in a cell line–specific manner suggests that additional biomarkers that predict the differential response are needed before any trials can be considered. To this end, we investigated the transcriptional profiles of R and OF to determine whether changes could help elucidate the mechanisms behind these pharmacological differences. We analyzed the transcriptomes of cell lines that showed favorable interactions using either R + chemo or OF + chemo. Because U2932 exhibited favorable interactions using R + chemo combinations and TMD8 showed favorable interactions using OF + chemo interactions, we analyzed the gene expression profiles induced by R and OF for these two cell lines. Four hours was chosen as the incubation time because it was the earliest time point at which differential pharmacological interactions were detected, thereby allowing us to identify the earliest transcriptional profiles that drive pharmacological differences (data not shown). To validate our RNA sequencing data, we measured the gene expression levels of eight randomly selected genes using qRT–PCR and compared them to those found using RNA sequencing (**Supplementary Figure 2**).

**Figure 4:**
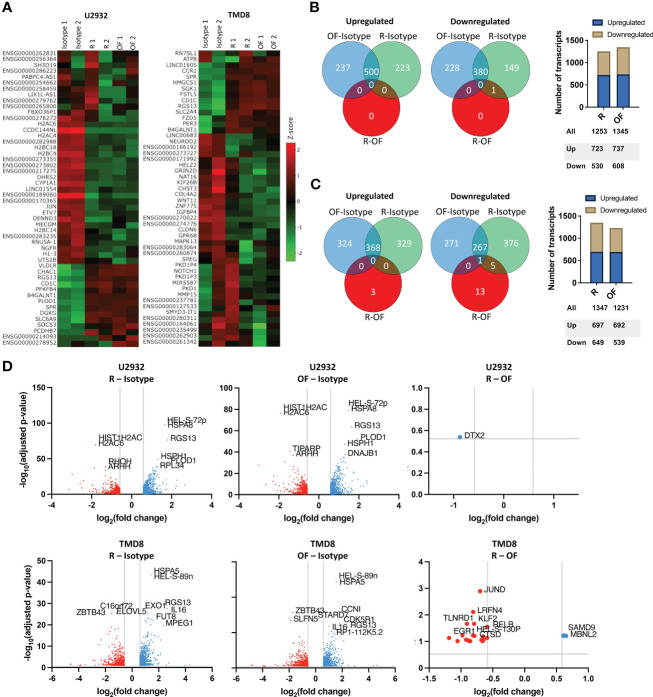
Rituximab and ofatumumab activate similar transcriptional profiles but differentially modulate a small subset of genes. **(A)** Heat map of the 50 most variable genes expressed by U2932 and TMD8 treated with R, OF, or IgG isotype control (20 µg/mL) for 4 hours. Venn diagrams and distribution of upregulated and downregulated genes in U2932 **(B)** and TMD8 **(C)**. **(D)** Volcano plots of differentially expressed genes between R and isotype, OF and isotype, and R and OF in U2932 and TMD8. The top 10 most significant differentially expressed genes were labeled in all graphs.

Fifty of the most variably expressed genes were hierarchically clustered on a heat map to visualize the global differences between cell lines treated with IgG isotype, R, or OF (**Figure 4A**). The heat maps show distinct gene expression profiles between cells treated with IgG isotype and anti-CD20 antibody but far smaller differences in gene expression profiles between cells treated with either R or OF.

Next, Venn diagrams of gene expression profiles were generated to quantify the number of upregulated and downregulated genes induced by R and OF (**Figures 4B, C**). In U2932, although both mAbs caused substantial changes compared to the isotype control, R and OF induced remarkably similar gene expression profiles compared to each other. R upregulated 723 genes and downregulated 530 genes, and OF upregulated 737 genes and downregulated 608 genes. There was only one differentially expressed gene between R and OF at a false discovery rate (FDR) of 0.3. For TMD8, R and OF induced substantial transcriptional changes compared to the isotype control and had more different gene expression profiles compared to each other. R upregulated 697 genes and downregulated 649 genes, and OF upregulated 692 genes and downregulated 539 genes. R preferentially upregulated 3 genes versus OF, whereas OF preferentially upregulated 19 genes versus R at an FDR = 0.10, a threshold used in previous cancer drug studies (41–46).

We then used volcano plots to show the top 10 most significant differentially expressed genes induced by R and OF (**Figure 4D**). In U2932, R preferentially downregulated Deltex E3 Ubiquitin Ligase 2 (DTX2) compared to OF. In TMD8, OF preferentially upregulated genes associated with apoptosis and chemosensitization (Synaptojanin 2 (SYNJ2), Cathepsin D (CTSD), TNF alpha induced protein 8 like 1 (TNFAIP8L1), and Chromosome 8 Open Reading Frame 82 (C8orf82)) and growth inhibition (transforming growth factor-beta 1 (TGFB1), Interleukin 4-induced gene-1 (IL4I1), and Kruppel-like transcription factor 2 (KLF2)) in DLBCLs compared to R. In summary, our data suggests that there is strong concordance in the gene expression profiles induced by R and OF, with subtle transcriptional differences between R and OF.

**Figure 5:**
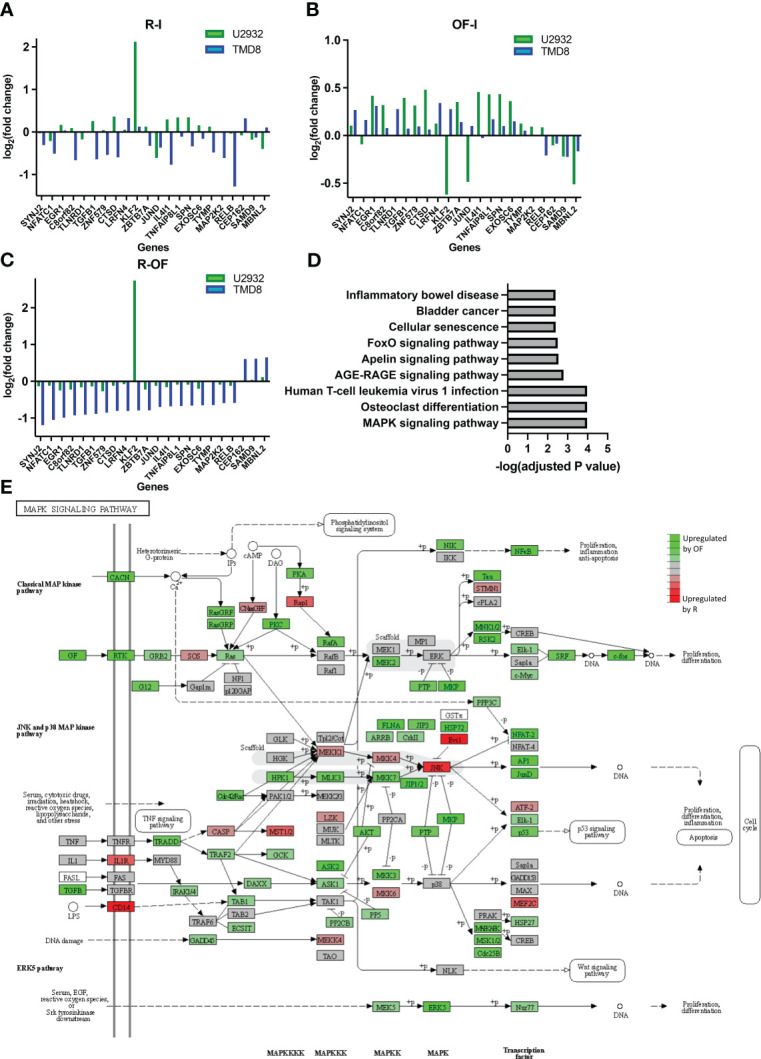
Ofatumumab differentially upregulates genes enriched in the JNK and p38 MAPK pathway over rituximab. **(A)** Fold changes of the significant differentially expressed genes modulated by R. **(B)** Fold changes of the significant differentially expressed genes modulated by OF. **(C)** Difference in fold changes of significant differentially expressed genes between the two anti-CD20s. **(D)** Gene enrichment analysis using the KEGG database. **(E)** These differences in TMD8 were mapped with related genes using the KEGG database to identify which specific family of the MAPK signaling pathway was most biologically relevant and, therefore, most likely involved in differential pharmacological interactions. Green represents genes that were preferentially upregulated by OF over R, whereas red represents genes preferentially upregulated by R over OF.

We next wanted to understand whether the directionality of these transcriptional differences between R and OF in TMD8 was cell line specific. We measured the fold changes of the 22 differentially expressed genes induced by R and OF in both TMD8 and U2932. In both cell lines, R preferentially downregulated these genes (**Figure 5A**), whereas OF preferentially upregulated these genes (**Figure 5B**). Interestingly, the directionality of these differences was conserved in 21 of the 22 genes across both cell lines (**Figure 5C**). Therefore, we conclude that these transcriptional differences between R and OF are conserved across cell lines.

Next, we proceeded to identify any biological pathways enriched within genes differentially expressed by R and OF. The KEGG is an integrated database resource consisting of curated databases manually mapped into molecular networks to biologically interpret sequencing data. We performed gene enrichment analysis using this database to identify enriched molecular pathways. Because of the remarkably similar gene expression changes induced by R and OF in U2932, we were unable to perform gene enrichment analysis for U2932. However, we found enrichment of nine pathways in TMD8 (**Figure 5D**). Among these pathways, the Mitogen-activated protein kinase (MAPK) pathway is the most significantly enriched and the most biologically relevant pathway, with previous studies showing that this pathway is modulated by R in sensitizing B-cell lymphomas to chemotherapy (47, 48). Therefore, our gene enrichment analysis suggests that R and OF may exert distinct effects through differential regulation of the MAPK signaling pathway.

Because the MAPK pathway comprises many families that modulate a broad spectrum of physiological processes, ranging from cell survival to apoptosis, it is important to identify which families of the MAPK pathway may be differentially modulated by R and OF (49). Therefore, using the KEGG database, we mapped differentially expressed genes with other known molecularly interacting genes to identify the most biologically relevant MAPK family (**Figure 5E**). Among the differentially modulated MAPK families, we find the c-Jun N-terminal kinases (JNK) and p38 MAPK pathway most biologically relevant to our findings for two reasons. First, we see that chemotherapy also upregulates this pathway. Second, previous studies have shown that upregulation of this pathway sensitizes DLBCLs to chemotherapy, such as vindesine and cyclophosphamide (50, 51). Therefore, these results may suggest that OF potentiates the cytotoxic and cytostatic potential of chemotherapy through complementary upregulation of the JNK and p38 MAPK signaling pathway, leading to enhanced downstream apoptosis and inhibition of proliferation.

## Discussion

Our work revealed vast heterogeneity in the pharmacological interactions within the R-CHOP regimen, illustrating the wide genetic and phenotypic heterogeneity expected of DLBCL. This finding may explain why some patients are cured by R-CHOP, whereas other patients relapse. We then demonstrated, for the first time, that two FDA-approved anti-CD20s can differentially potentiate CHOP in a cell line–specific manner. The observed difference between R and OF was unexpected because they have similar direct killing capacities *in vitro* and were comparable in their clinical efficacy in phase III clinical trials (18–21). For this reason, these findings reveal novel mechanisms of different anti-CD20 mAbs independent of their ability to modulate immune function. Although we observe that R and OF preferentially synergize with chemotherapy in a cell line–specific manner, we also observe that these anti-CD20s generally synergize with chemotherapy more in ABC DLBCLs and antagonize chemotherapy more in GCB DLBCLs. This paradoxical role of these anti-CD20s could possibly be explained by the fact that R is known to simultaneously activate pro-survival pathway Akt, which drives lymphomagenesis in GCB, and to downregulate pro-survival pathway NF-kB, which drives lymphomagenesis in ABC but not in GCB (52–54).

Our initial findings of the pharmacological interactions between R and CHOP validated previous studies that showed that R potentiates some chemotherapies (35). R potentiated cytotoxic chemotherapy drugs such as O and cytostatic properties of glucocorticoids such as P. However, our findings also demonstrated that R can antagonize chemotherapy in some cell lines, as we observed in SUDHL2 and SUDHL4. This finding is unexpected because R has been characterized as a general chemotherapy potentiator in the field. It is possible that this interaction could advise the use R and chemotherapy concurrently, pre-administration, or post-administration of chemotherapy to avoid an unfavorable immunochemotherapy interaction, which is a recognized important question in the field (55). Last, we characterized interactions between CDC and chemotherapy in R-CHOP, which remained largely unexplored (34).

We also compared the transcriptional profiles of DLBCLs bound by R and OF to uncover potential mechanisms for these pharmacological differences. We found that, compared to R, OF preferentially upregulates genes enriched in the p38 MAPK pathway, leading to downstream apoptosis and growth inhibition. This transcriptional difference could mechanistically explain how R and OF differentially potentiate chemotherapy for two reasons. First, on the basis of KEGG analysis, chemotherapy upregulates this same pathway, and, therefore, potentiated killing and growth inhibition may occur through functional complementation. In addition, a previous study found that an organic molecule activating p38 MAPK synergized with vinblastine and cyclophosphamide to kill DLBCLs (50). Interestingly, previous studies show that R sensitizes B-cell lymphomas to chemotherapy through inhibition, rather than upregulation of p38 MAPK, suggesting that R and OF may sensitize DLBCLs to chemotherapy through opposite modulation of p38 MAPK (24, 47).

R and OF were presumed to have similar direct signaling activities, based on previous studies and our findings (39). However, our results from *in vitro* experiments found that these two mAbs that bind to different epitopes on CD20 can initiate different downstream mechanisms that potentiate different classes of chemotherapy. A previous report showed that R and a non-clinical anti-CD20 mAb induced different transcriptional profiles (56). Therefore, it is conceivable that targeting other CD20 epitopes could elicit different pathways and activate favorable immunochemotherapy interactions across a broader range of DLBCLs. These findings may advise a new design strategy for new anti-CD20s that focus on targeting different epitopes of CD20 to expand favorable immunochemotherapy interactions across a greater range of DLBCLs. This strategy is particularly clinically relevant since the expiration of the R patent in 2016, because there has been a breakthrough in other anti-CD20 biologics such as ocrelizumab, ublituximab, and obinutuzumab, which are already approved for use in patients. Given the usage of anti-CD20s in nearly all B-cell pathologies, the findings of this paper may also have potential therapeutic value in a broad range of B-cell–related diseases including multiple sclerosis, chronic lymphocytic leukemia, rheumatoid arthritis, Burkitt leukemia, and others.

Further studies will be needed to address limitations of our findings and test their potential implications. In this study, we scored the pharmacological interactions between R and chemotherapy and between CDC and chemotherapy. However, R is cytotoxic to DLBCL by ADCC and ACP, and we cannot exclude that these mechanisms could also interact with chemotherapy. Indeed, there has been a study showing that dexamethasone enhances R-mediated ADCC through increased phosphatidylserine exposure on DLBCLs (34). Therefore, studying the pharmacological interactions between cellular-mediated effects and chemotherapy could elucidate additional immunochemotherapy interactions specific to the *in vivo* setting.

Our transcriptome analysis reveals a mechanistically plausible finding explaining the pharmacological differences between R and OF, validated by correlating gene fold expression using qRT-PCR (**Supplementary Figure 1**). However, DLBCL is characterized by a notable degree of biological heterogeneity, and our analysis is limited to a few select cell lines. Therefore, we present our transcriptional analysis as hypothesis-generating, rather than conclusive across all DLBCLs. Furthermore, the direct effects of anti-CD20 are known to activate direct signaling and transcriptional pathways, necessitating further investigation through a multi-omics approach (57). Ultimately, whole-exome, transcriptomic, and phosphoproteomic analysis would provide additional mechanistic insight into these findings and elucidate potential baseline biomarkers predicting favorable outcomes using either R or OF.

## Data availability statement

The datasets presented in this study can be found in online repositories. The names of the repository/repositories and accession number(s) can be found below: GEO, accession number GSE228687.

## Author contributions

BL: conceptualization, method design, data analysis, experimentation, validation, writing, and funding acquisition. TP: conceptualization, method design, data analysis, experimentation, validation, writing, and funding acquisition. AA: conceptualization, method design, data analysis, writing, funding acquisition, methodology, project administration, resources, and supervision. KR: conceptualization, method design, data analysis, writing, funding acquisition, methodology, project administration, resources, and supervision. All authors contributed to the article and approved the submitted version.
